# Toxoplasmosis-Related Knowledge and Practices Among Pregnant Women in the United States

**DOI:** 10.1080/10647440300025512

**Published:** 2003

**Authors:** Jeffrey L. Jones, Folashade Ogunmodede, Joni Scheftel, Elizabeth Kirkland, Adriana Lopez, Jay Schulkin, Ruth Lynfield

**Affiliations:** 1 Division of Parasitic Diseases National Center for Infectious Diseases Centers for Disease Control and Prevention Mailstop F-22, 4770 Buford Highway NE Atlanta GA 30341-3724 USA; 2 Acute Disease Investigation and Control Section Minnesota Department of Health Minneapolis MN USA; 3 Department of Research American College of Obstetricians and Gynecologists Washington, DC USA

## Abstract

*Background:* Infection with *Toxoplasma gondii*  during pregnancy can lead to severe illness in the fetus. Many *T. gondii* infections are preventable by simple hygienic measures.

*Methods:* We surveyed pregnant women in the US to determine their knowledge about toxoplasmosis and their practices to prevent infection. Volunteer obstetricians selected to be demographically representative of the American College of Obstetricians and Gynecologists recruited the participants.

*Results:* Of 403 women responding to the survey, 48% indicated that they had heard or seen information about toxoplasmosis; however, only 7% were aware of being tested for the disease. Forty percent of responding women knew that toxoplasmosis is caused by an infection, but 21% thought that a poison causes it. The highest level of knowledge was about cats and *T. gondii* ; 61% responded that the organism is shed in the feces of infected cats and 60% responded that people could acquire toxoplasmosis by changing cat litter. There was a low level of knowledge about other risk factors; only 30% of the women were aware that *T. gondii*  may be found in raw or undercooked meat. Nevertheless, a high percentage of women indicated that they do not eat undercooked meat during pregnancy and that they practice good hygienic measures such as washing their hands after handling raw meat, gardening or changing cat litter.

*Conclusion:* Except for the risk of transmission from cats, knowledge among pregnant women about toxoplasmosis is low. However, toxoplasmosis-preventive practices are generally good, suggesting that providers should continue to offer education about practices that help prevent foodborne diseases in general as well as information about preventing toxoplasmosis specifically.


*Infect Dis Obstet Gynecol 2003;11:139–145*

### Toxoplasmosis-related knowledge and practices among pregnant women in the United States

**Jeffrey L. Jones[1], Folashade Ogunmodede[2], Joni Scheftel[2], Elizabeth Kirkland[1], Adriana Lopez[1], Jay Schulkin[3] and Ruth Lynfield[2]**

[1]*Division of Parasitic Diseases, National Center for Infectious Diseases, Centers for Disease Control and Prevention, Atlanta, GA*
[2]*Acute Disease Investigation and Control Section, Minnesota Department of Health, Minneapolis, MN*
[3]*Department of Research, American College of Obstetricians and Gynecologists, Washington, DC*

***Background:*** Infection with *Toxoplasma gondii* during pregnancy can lead to severe illness in the fetus. Many *T. gondii* infections are preventable by simple hygienic measures.
***Methods:*** We surveyed pregnant women in the US to determine their knowledge about toxoplasmosis and their practices to prevent infection. Volunteer obstetricians selected to be demographically representative of the American College of Obstetricians and Gynecologists recruited the participants.
***Results:*** Of 403 women responding to the survey, 48% indicated that they had heard or seen information about toxoplasmosis; however, only 7% were aware of being tested for the disease. Forty percent of responding women knew that toxoplasmosis is caused by an infection, but 21% thought that a poison causes it. The highest level of knowledge was about cats and *T. gondii*; 61% responded that the organism is shed in the feces of infected cats and 60% responded that people could acquire toxoplasmosis by changing cat litter. There was a low level of knowledge about other risk factors; only 30% of the women were aware that *T. gondii* may be found in raw or undercooked meat. Nevertheless, a high percentage of women indicated that they do not eat undercooked meat during pregnancy and that they practice good hygienic measures such as washing their hands after handling raw meat, gardening or changing cat litter.
***Conclusion:*** Except for the risk of transmission from cats, knowledge among pregnant women about toxoplasmosis is low. However, toxoplasmosis-preventive practices are generally good, suggesting that providers should continue to offer education about practices that help prevent foodborne diseases in general as well as information about preventing toxoplasmosis specifically.

Key words: PREVENTION; SURVEY; RISK FACTORS; TOXOPLASMOSIS

---

Infection with *Toxoplasma gondii* during pregnancy can lead to severe illness in the fetus and infant including central nervous system and ocular disease, and even death[1]. Based on local and regional studies, the Centers for Disease Control and Prevention (CDC) estimates that there are 400–4000 cases of congenital toxoplasmosis per year[2] and that toxoplasmosis is the third leading cause of foodborne deaths in the US[3]. Population-based studies in the US have shown that most (85%) pregnant women are susceptible to infection with *T. gondii*[4]. Many congenital *T. gondii* infections acquired during gestation are preventable by simple precautions during

Correspondence to: Jeffrey L. Jones, MD, MPH, Division of Parasitic Diseases, Centers for Disease Control and Prevention, NCID, Mailstop F-22, 4770 Buford Highway NE, Atlanta, GA 30341-3724, USA. Email: jlj1@cdc.gov

© 2003 The Parthenon Publishing Group 

pregnancy. These precautions include fully cooking meat, not ingesting uncooked or undercooked meat and not ingesting soil or water contaminated with cat feces (for example, soil from sand boxes and cat litter boxes, or soil on raw fruits and vegetables[2]).

In order to determine their knowledge about toxoplasmosis and the practices that prevent infection, the Department of Research of the American College of Obstetricians and Gynecologists (ACOG), in collaboration with the CDC and the Minnesota Department of Health (MDH), surveyed pregnant women in the US. We present here the results of the survey and a discussion of congenital toxoplasmosis prevention.

### METHODS

In the fall of 2002, the Department of Research of ACOG asked physician members of its Collaborative Ambulatory Research Network (CARN) to participate in the survey. The members of the network are practicing obstetrician-gynecologists who volunteer to participate in surveys implemented by the College. CARN members are chosen to be demographically representative of all ACOG members by age and gender. The study was limited to obstetrician-gynecologists practicing in the US. Participating physicians were asked to recruit up to five volunteer pregnant women from their practice to complete the survey.

The MDH developed the survey with input from staff at the CDC and ACOG. Infectious disease physicians, public health physicians, health educators and laboratorians assisted in the development of the questionnaire. Demographic information was requested, followed by questions covering general knowledge about toxoplasmosis, risk factors, symptoms and timing of infection, prevention knowledge and preventive behavior. The questionnaire took approximately 20 minutes to complete. Pilot testing was done at CDC (among non-medical staff) and by ACOG obstetricians. No personal identifiers were recorded in the survey. The survey was reviewed and exempted by the MDH, ACOG and CDC institutional human subjects review boards.

The survey data were analyzed using techniques for random samples. Ninety-five percent confidence limits (CL) were calculated for proportions; the maximum 95% CL are given in a footnote to Table 2 (i.e. the maximum 95% CL calculated for any question). Because we collected zip codes and felt that respondents may be more similar if they were from the same zip code area, we also analyzed the data as if it were collected by a one-stage cluster design with zip code being the primary sampling unit. Of the 327 unique zip codes recorded in the survey, 287 (87.8%) had one respondent, 31 (9.5%) had two respondents, 6 (1.8%) had three respondents and 3 (0.9%) had four respondents. When the data were analyzed as a one-stage cluster design, the design effect was less than 1.2 indicating that clustering of responses by zip code did not have a large effect on the variance. Nonetheless, we used 1.2 as a design effect when calculating the maximum 95% CL shown in the footnote to Table 2 (Clopper–Pearson method, StatXact 5 software[5]). The stratified analysis was conducted with EpiInfo[6] software. Trends for categorical variables were analyzed with the chi-square for trend and differences between categorical variables were analyzed with the chi-square test.

### RESULTS

Two hundred twenty-five CARN physician members participated in the survey and recruited 403 pregnant women. The median age of the pregnant women surveyed was 29 (range 12–49), the median duration of pregnancy was 30 weeks (range 1–40) and the median parity was 1 (range 0–6); other characteristics of the study population are shown in Table 1. Information was not collected on the number of pregnant women who declined to participate. Respondents were well dispersed throughout the US by region, but were more likely to be white, educated and live in rural locations than the general US population (Table 1).

Knowledge about toxoplasmosis and how to prevent it varied by topic, but in general there was a great deal of uncertainty about the disease (Table 2). Only 7% of respondents indicated that they had been tested for toxoplasmosis; however, 43% of respondents were uncertain if they had

**Table 1** Characteristics of pregnant women surveyed in the US, 2002, $n = 403$

| *Demographics* | *Study population* (%) | *US population** (%) |
|---|---|---|
| Education** | | |
| < High school graduate | 1 | 11 |
| High school graduate | 17 | 31 |
| Some college | 28 | 22 |
| College graduate | 37 | 29 |
| Graduate school | 17 | 7 |
| Residence location*** | | |
| Rural | 33 | 20 |
| Suburban | 53 | 50 |
| Urban | 15 | 30 |
| Race | | |
| American Indian/ Alaskan native | < 1 | < 1 |
| Asian or Pacific Islander | 5 | 4 |
| Black | 9 | 12 |
| White | 79 | 75 |
| Other | 7 | 8 |
| Ethnicity | | |
| Hispanic | 9 | 13 |
| US region | | |
| Northeast | 17 | 19 |
| South | 39 | 36 |
| Midwest | 28 | 23 |
| West | 17 | 22 |

*2000 Census, $n = 281\,421\,906$; **for US population, education for women 20–49 years of age, $n = 61\,219\,000$; ***for US population, categories are: central city, metropolitan statistical area (MSA) outside of central city, and non-MSA

been tested. Only 40% of respondents were certain that toxoplasmosis is caused by an infection and 21% thought that a poison causes it. The highest level of knowledge was about cats and toxoplasmosis, 61% indicated that toxoplasmosis (*T. gondii*) is shed in the feces of infected cats and 60% indicated that people can be exposed to toxoplasmosis by changing cat litter. Respondents also seemed to be reasonably aware that pregnant women can develop serious complications after acquiring toxoplasmosis (58%) and that unborn and newborn children can develop serious complications from toxoplasmosis (56%).

There was a low level of knowledge about risk factors for toxoplasmosis other than cat feces, as noted by the following examples: only 30% of respondents indicated that the organism that causes toxoplasmosis may be found in raw or undercooked meat, 26% indicated that people can get toxoplasmosis by eating undercooked pork and 29% indicated that people can acquire toxoplasmosis by gardening without gloves. In general, respondents were unsure of the answers to questions about the timing of infection and the symptoms of toxoplasmosis in both the mother and the infant (Table 2, symptoms and timing of infection section). In addition, except for avoiding changing the cat litter box during pregnancy, there was uncertainty about ways to avoid or prevent toxoplasmosis (Table 2, prevention knowledge section).

When we examined key questions about toxoplasmosis, knowledge often increased with higher levels of education and less frequently with age (Table 3). However, there was not as much variation in the level of knowledge by trimester of pregnancy or parity (first pregnancy versus two or more). The numbers were quite small in many racial/ethnic categories, therefore we split the racial/ethnic groups into two categories: (i) white-nonHispanic and (ii) non-white or Hispanic. When examining the key questions in Table 3 by race/ethnicity, a higher percentage of white-nonHispanic persons than non-white or Hispanic persons had heard of toxoplasmosis (51% versus 36%, $p = 0.008$), knew that toxoplasmosis can be prevented by cooking meat well (52% versus 37%, $p = 0.015$), knew that people can get toxoplasmosis by gardening without gloves (34% versus 17%, $p = 0.002$), knew that toxoplasmosis can be avoided by only feeding cats dry or commercial cat food and not letting them kill and eat rodents (34% versus 17%, $p = 0.002$) and knew that toxoplasmosis can be prevented by thoroughly washing and/or peeling all fruits and vegetables before eating them (44% versus 29%, $p = 0.014$). There were no significant differences by region of the country.

Although in many topic areas there was a lack of knowledge about toxoplasmosis, its symptoms and how it is prevented, the women surveyed indicated that they practice behaviors that would prevent toxoplasmosis. Of the women responding, 93% indicated that they routinely wash their hands after gardening, 80% routinely washed their hands after changing cat litter, 96% routinely washed

**Table 2** Toxoplasmosis-related knowledge and preventive practices of pregnant women in the US, 2002, $n = 403$*

| Questions | *n* | Yes (%) | No (%) | Not sure (%) |
|---|---|---|---|---|
| *General information and knowledge* | | | | |
| Have you ever read, heard or seen any information about toxoplasmosis (see text for where information acquired)? | 403 | 48 | 43 | 8 |
| Have you ever been tested for toxoplasmosis? | 402 | 7 | 50 | 43 |
| Is toxoplasmosis caused by an infection? | 395 | 40** | 13 | 47 |
| Is toxoplasmosis caused by a poison? | 389 | 21 | 31** | 49 |
| Is toxoplasmosis (*Toxoplasma gondii*) shed in the feces of infected cats? | 390 | 61** | 1 | 39 |
| Is toxoplasmosis (*T. gondii*) sometimes found in raw or undercooked meat? | 388 | 30** | 15 | 56 |
| *Risk factors* | | | | |
| Can people get toxoplasmosis by changing cat litter? | 396 | 60** | 3 | 37 |
| Can people get toxoplasmosis by eating undercooked pork? | 393 | 26** | 21 | 52 |
| Can people get toxoplasmosis by handling raw venison (deer meat)? | 391 | 22** | 17 | 61 |
| Can people get toxoplasmosis by gardening without gloves? | 390 | 29** | 20 | 59 |
| *Symptoms and timing of infection* | | | | |
| Can pregnant women develop serious complications after infection with toxoplasmosis (*T. gondii*)? | 397 | 58** | 4 | 39 |
| Can unborn and/or newborn children develop serious complications after infection with toxoplasmosis (*T. gondii*)? | 393 | 56** | 3 | 42 |
| Can toxoplasmosis in a pregnant woman cause fever and feeling like you have the 'flu'? | 398 | 36** | 9 | 62 |
| Can toxoplasmosis in a pregnant woman cause swollen glands (lymph nodes)? | 395 | 25** | 5 | 70 |
| Can toxoplasmosis in a pregnant woman cause no symptoms? | 395 | 17** | 16 | 68 |
| Toxoplasmosis (*T. gondii*) can only be passed from a pregnant woman to her fetus if she is newly infected during that pregnancy. | 396 | 28** | 5 | 68 |
| Toxoplasmosis (*T. gondii*) is rarely passed from a pregnant woman to her fetus if she was infected before becoming pregnant. | 385 | 15** | 13 | 73 |
| A baby with toxoplasmosis may have no signs of illness at birth, but develop illness later. | 387 | 20** | 5 | 75 |
| A baby with toxoplasmosis may have vision problems | 385 | 20** | 2 | 78 |
| A baby with toxoplasmosis may be treated with medicine | 387 | 27** | 3 | 71 |
| *Prevention knowledge* | | | | |
| Ways to avoid toxoplasmosis include: feeding your cat dry or commercial cat food and not letting it kill and eat rodents. | 387 | 29** | 11 | 60 |
| Ways to avoid toxoplasmosis include: avoiding stray cats. | 387 | 47** | 53 | 47 |
| Ways to avoid toxoplasmosis include: letting someone else change the cat's litter box. | 391 | 65** | 1 | 34 |
| Ways to avoid toxoplasmosis include: making sure the cat's litter box is changed daily. | 388 | 45** | 8 | 47 |
| Toxoplasmosis can be prevented by cooking meat well until no pink is seen and the juices run clear. | 387 | 48** | 3 | 50 |
| Toxoplasmosis can be prevented by thoroughly washing and/or peeling all fruits and vegetables before eating them. | 388 | 39** | 5 | 56 |
| Toxoplasmosis may be prevented by cleaning all cutting boards and utensils thoroughly after each use. | 389 | 49** | 1 | 50 |
| *Preventive behavior* | | | | |
| Since becoming pregnant, do you routinely wash your hands after gardening? | 388 | 93 | 3 | 4 |
| Since becoming pregnant, do you routinely wash your hands after changing cat litter? | 298 | 80 | 12 | 8 |
| Since becoming pregnant, do you routinely wash your hands after handling raw meat? | 396 | 96 | 2 | 3 |
| Since becoming pregnant, do you eat rare meat? | 397 | 6 | 92 | 2 |

*The number of respondents may vary for some questions due to non-response. Percentages read horizontally may not add to 100% due to rounding. Ninety-five percent confidence limits for percentages are ± 6.7% or less (see Methods). **Correct answer for knowledge questions their hands after handling raw meat and 92% indicated that they had not eaten rare meat during pregnancy.

Of the 403 women who participated in the survey, 192 (48%) indicated that they had heard or seen information about toxoplasmosis. On the

**Table 3** Selected questions about toxoplasmosis stratified by age, education, trimester and number of pregnancies

| | *Age (years) % correct* | | | *Education % correct* | | | | *Trimester % correct* | | | *Pregnancy % correct* | |
|---|---|---|---|---|---|---|---|---|---|---|---|---|
| *Question* | *≤ 25* | *26–34* | *≥ 35* | *≤ High school* | *Some college* | *College graduate* | *Graduate school* | *1st* | *2nd* | *3rd* | *1st* | *≥ 2* |
| Have you ever read, heard or seen any information about toxoplasmosis? | 39 | 53 | 50 | **26*** | **46** | **54** | **66** | 40 | 46 | 51 | 48 | 49 |
| Is toxoplasmosis caused by an infection? | **34** | **40** | **49** | **24** | **36** | **44** | **51** | 38 | 37 | 42 | 39 | 39 |
| Can people get toxoplasmosis by eating undercooked pork? | 27 | 26 | 24 | 25 | 27 | 27 | 23 | 17 | 29 | 27 | **33** | **22** |
| Is toxoplasmosis (*T. gondii*) sometimes found in raw or undercook meat? | 26 | 31 | 29 | 21 | 30 | 31 | 30 | 27 | 33 | 28 | 36 | 25 |
| Toxoplasmosis can be prevented by cooking meat well until no pink is seen and the juices run clear. | **36** | **50** | **58** | **29** | **41** | **54** | **67** | 47 | 49 | 48 | 52 | 45 |
| Can people get toxoplasmosis by gardening without gloves? | **14** | **35** | **33** | **10** | **26** | **35** | **44** | 26 | 24 | 32 | 31 | 29 |
| Usually toxoplasmosis can only be passed from a pregnant women to her fetus if she is newly infected during that pregnancy | **20** | **27** | **37** | **19** | **26** | **27** | **41** | 28 | 24 | 29 | 26 | 29 |
| Ways to avoid toxoplasmosis include feeding your cat dry or commercial cat food and not letting it kill and eat rodents | 26 | 33 | 26 | **17** | **25** | **33** | **42** | 27 | 32 | 29 | 35 | 25 |
| Toxoplasmosis can be prevented by thoroughly washing and/or peeling all fruits and vegetables before eating them | 32 | 43 | 40 | **21** | **35** | **46** | **53** | 47 | 38 | 39 | 46 | 36 |

*Significant trends or differences in bold for age, education, trimester or number of pregnancies (read horizontally, chi-square or chi-square for trend, $p < 0.05$)

survey form, respondents were asked to check all information sources where they had heard about toxoplasmosis (multiple answers were accepted, so responses total greater than 100%). Of these 192 women, 137 (71%) indicated that they had heard about toxoplasmosis from magazines or books on pregnancy and childbirth, 103 (53%) from a medical professional, 86 (45%) from friends or family, 27 (13%) from childbirth classes or health classes and less than 1% from a government agency or television.

### DISCUSSION

This national survey of pregnant women about toxoplasmosis documents a relatively low level of knowledge about the disease, symptoms and how to prevent infection. The highest level of knowledge was about the association between cats and toxoplasmosis. There was a relatively low level of knowledge about the role of undercooked meat and other risk factors for *T. gondii* infection. The United States Department of Agriculture estimates that one-half of the *T. gondii* infections in the US are due to ingestion of undercooked meat[7]. A recent community-based case–control study supports this estimate[8].

It is interesting that knowledge about toxoplasmosis did not vary greatly by trimester of pregnancy. It may be that efforts to educate pregnant women about toxoplasmosis occur most often in the first trimester, so knowledge does not increase in subsequent trimesters. For key knowledge questions, the results also varied by race–ethnicity, suggesting an even more important role of preventive education for some racial and ethnic groups.

The findings of this study indicate that health professionals play an important role in informing women about toxoplasmosis. Of those women who had heard about toxoplasmosis, over half had heard about it from a medical professional. A previous national survey found that US

obstetrician-gynecologists are well informed about how to prevent toxoplasmosis[9]. The findings of this study also indicate that women's magazines and books are a good place to reach women with information about toxoplasmosis.

Our survey is subject to several limitations. The physicians and patients participating in the survey were volunteers, therefore the results are not representative of all pregnant women in the US and may be subject to some statistical biases. The women in our study were more highly educated than women in the US population (Table 1) and because we found that toxoplasmosis-related knowledge increased with educational level, our findings may over-estimate knowledge about this disease among US women. In addition, because a very high percentage of women indicated that they practice good preventive behaviors, one wonders if the respondents were motivated to give the correct answers because the survey was provided in their place of medical care. However, most women (in general, not specifically pregnant women) also responded that they practice good preventive behaviors in a survey of the general population about foodborne disease conducted by the CDC in 1998–1999[10].

It is encouraging that a study in Canada has shown that even brief education of pregnant women helps to improve some congenital toxoplasmosis preventive behaviors[11] and that a study in Belgium found that health education was associated with a 63% reduction in *T. gondii* seroconversion[12]. In addition, researchers working in Poland found that toxoplasmosis-related education of pregnant women doubled their knowledge about the disease and prevention in 4 years[13].

Education about meat- and cat/soil-related hygiene should be provided to women of childbearing age by educational institutions and to pregnant women at their first prenatal visit[2]. It is important that the educational materials be complete and accurate[14] and that they be made available in a culturally and linguistic appropriate format[15]. With these measures, many cases of congenital toxoplasmosis could be prevented.

### REFERENCES

1. Remington JS, McLeod R, Thulliez P, *et al.* Toxoplasmosis. In Remington JS, Klein JO, eds. *Infectious Diseases of the Fetus and Newborn Infant*, 5th edn. Philadelphia: Saunders, 2001: 205–346
2. Centers for Disease Control and Prevention. CDC recommendations regarding selected conditions affecting women's health. *MMWR Morb Mortal Wkly Rep* 2000;49:57–75
3. Mead PS, Slutsker L, Dietz V, *et al.* Food-related illness and death in the United States. *Emerg Infect Dis* 1999;5:607–24
4. Jones JL, Kruszon D, Wilson M, *et al. Toxoplasma gondii* infection in the United States: seroprevalence and risk factors. *Am J Epidemiol* 2001; 154:357–65
5. Stat Xact ver. 5.03, Cytel Software Corp., Cambridge, MA
6. Dean AG, Dean JA, Coulombier D, *et al.* Epi Info, Version 6: A Word Processing, Database, and Statistics Program for Epidemiology on Microcomputers. Centers for Disease Control and Prevention, Atlanta, GA, 1994
7. Buzby JC, Roberts T. ERS updates U.S. foodborne disease costs for seven pathogens. *Food Review* (Sept–Dec) 1996;19:20–5
8. Roghmann MC, Faulkner CT, Lefkowitz A, *et al.* Decreased seroprevalence for *Toxoplasma gondii* in Seventh Day Adventists in Maryland. *Am J Trop Med Hyg* 1999;60:790–2
9. Jones JL, Dietz VJ, Power M, *et al.* Survey of obstetrician-gynecologists in the United States about toxoplasmosis. *Infect Dis Obstet Gynecol* 2001;9:23–31
10. Centers for Disease Control and Prevention. Foodborne Diseases Active Surveillance Network (FoodNet): Population Survey of Exposures: 1998–1999. Atlanta: Centers for Disease Control and Prevention, 1999
11. Carter AO, Gelmon SB, Wells GA, Toepell AP. The effectiveness of a prenatal education programme for the prevention of congenital toxoplasmosis. *Epidemiol Infect* 1989;103:539–45
12. Foulon W, Naessens A, Derde MP. Evaluation of the possibilities for preventing congenital toxoplasmosis. *Am J Perinatol* 1994;11:57–62

13. Pawlowski ZS, Gromadecka-Sutkiewicz M, Skommer J, *et al*. Impact of health education on knowledge and prevention behavior for congenital toxoplasmosis: the experience in Poznan, Poland. *Health Educ Res* 2001;16:493–502
14. Newton LH, Hall SM. A survey of health education material for the primary prevention of congenital toxoplasmosis. *Commun Dis Rep CDR Rev* 1995;5:R21–7
15. Jara M, Hsu HW, Eaton RB, Demaria A Jr. Epidemiology of congenital toxoplasmosis identified by population-based newborn screening in Massachusetts. *Pediatr Infect Dis J* 2001;20: 1132–5

RECEIVED 05/05/03; ACCEPTED 07/30/03
